# Influence of the Depth of the Convolutional Neural Networks on an Artificial Intelligence Model for Diagnosis of Orthognathic Surgery

**DOI:** 10.3390/jpm11050356

**Published:** 2021-04-29

**Authors:** Ye-Hyun Kim, Jae-Bong Park, Min-Seok Chang, Jae-Jun Ryu, Won Hee Lim, Seok-Ki Jung

**Affiliations:** 1 Department of Orthodontics, School of Dentistry, Dental Research Institute, Seoul National University, Seoul 03080, Korea; seoulclear@gmail.com (Y.-H.K.); dashers@hanmail.net (M.-S.C.); 2 Department of Oral and Maxillofacial Surgery, School of Dentistry, Seoul National University, Seoul 03080, Korea; bellagio@snu.ac.kr; 3 Department of Prosthodontics, Korea University Anam Hospital, Seoul 02841, Korea; kopros@korea.ac.kr; 4 Department of Orthodontics, Korea University Guro Hospital, Seoul 08308, Korea

**Keywords:** artificial intelligence, deep learning, orthognathic surgery diagnosis, convolutional neural network, cephalometric analysis

## Abstract

The aim of this study was to investigate the relationship between image patterns in cephalometric radiographs and the diagnosis of orthognathic surgery and propose a method to improve the accuracy of predictive models according to the depth of the neural networks. The study included 640 and 320 patients requiring non-surgical and surgical orthodontic treatments, respectively. The data of 150 patients were exclusively classified as a test set. The data of the remaining 810 patients were split into five groups and a five-fold cross-validation was performed. The convolutional neural network models used were ResNet-18, 34, 50, and 101. The number in the model name represents the difference in the depth of the blocks that constitute the model. The accuracy, sensitivity, and specificity of each model were estimated and compared. The average success rate in the test set for the ResNet-18, 34, 50, and 101 was 93.80%, 93.60%, 91.13%, and 91.33%, respectively. In screening, ResNet-18 had the best performance with an area under the curve of 0.979, followed by ResNets-34, 50, and 101 at 0.974, 0.945, and 0.944, respectively. This study suggests the required characteristics of the structure of an artificial intelligence model for decision-making based on medical images.

## 1. Introduction

Diagnosis is the definition of a patient’s problem; treatment planning is the process of eliminating the problem [1]. If the clinician does not properly diagnose a patient’s skeletal problems and simply performs orthodontic treatment to improve the dentition, the patient’s fascial profile may worsen. Therefore, it is first necessary to accurately diagnose a patient’s problem using various diagnostic clinical data [2,3]. Whether orthognathic surgery is necessary or a compromise orthodontic treatment is feasible is an important issue for dental patients who visit a hospital for treatment [1]. Orthognathic surgery can be effective, but it requires general anesthesia and is expensive and risky. Therefore, patients tend to prefer orthodontic treatment. However, there are cranial problems that cannot be resolved with orthodontic treatment alone. Prominent jaw, retruded mandible, and jaw asymmetry can only be corrected with orthognathic surgery. Orthognathic surgery is also chosen when there is a limit to the esthetic improvement through orthodontic treatment. Clinicians should consider orthognathic surgery if it is impossible to achieve adequate occlusion with orthodontic treatment alone, or if it is impossible to resolve the patient’s chief complaint. In order to maximize the effect of orthognathic surgery, it is necessary to plan appropriate presurgical orthodontic treatment and correct dental compensations. In many cases, pre-surgical orthodontic treatment proceeds in the opposite direction to camouflage orthodontic treatment. For example, an irreversible plan, such as extraction, should not be determined prematurely without the determination that orthognathic surgery is necessary. Therefore, deciding on whether orthognathic surgery is needed is the most important decision when establishing a treatment plan. Along with various clinical data, the clinician’s judgment plays an important role in identifying patients’ needs and establishing treatment plans. These surgery decisions may vary between clinicians due to differences in experience with procedures; in particular, clinicians with limited experience have difficulty making such judgments. As currently there is no standardized criterion for decision-making regarding the need for orthognathic surgery, a predictive statistical model or method is needed to support such decision-making [4,5,6]. The process of establishing a treatment plan by an expert is a process involving a wide variety of diagnostic data, background knowledge, and clinician experience, which are comprehensively and elaborately organized so that they cannot be formulated using a kind of formula. A system that imitates this judgment process will be of great help to inexperienced orthodontists. For example, a system wherein diagnostic values are input to a built artificial intelligence system, and the output is the treatment plan.

There are two important and irreversible decisions in orthodontics. One is deciding which tooth to extract and the other is whether to perform orthognathic surgery [7,8,9,10]. The most important factor in such decisions is to identify a patient’s skeletal problem and this diagnosis is made by identifying the patient’s skeletal pattern using cephalometric radiographs. In determining whether the orthognathic surgery is necessary; whether the difference between the maxillary and mandible can be overcome only with orthodontic treatment is also important.

In the past, an anatomical landmark was traced manually on a cephalometric radiograph and the measurements were numerically analyzed as the basis for a decision. When considering orthognathic surgery in the treatment plan, a surgical treatment objective (STO) or a visual treatment objective (VTO) should be determined in advance to specifically plan the direction of orthognathic treatment and the method of orthognathic surgery. At this time, the analysis of the measurements of the lateral cephalometric radiograph are helpful in establishing presurgical correction and an orthognathic surgery plan for skeletal malocclusion patients. Various measurement values quantitatively express the location of the jaw, teeth, and soft tissue, providing a criterion for each suitable location. In general, about 50 landmarks are used for a lateral cephalogram; various measurements, such as the length and angle of a line using these landmarks, are used for judgment. Each clinician has their own opinion on which measurements are important and thus make judgments in light of their own experience. However, measurements may also be unreliable based on the value. Even the SNA angle and SNB angle, which are measurements of the anterior and posterior positions of the maxilla and mandible, may have different values even in the same skeletal pattern due to changes in the position of the nasion.

The development of artificial intelligence has profoundly impacted image analysis, particularly the analysis of medical images [11,12,13,14]. In image classification, the advances in deep learning has been remarkable and, in 2015, the ImageNet Large Scale Visual Recognition Challenge (ILSVRC) went beyond human perception. Since dentistry is no exception, many algorithms have been developed that automatically detect these anatomical landmarks using diverse artificial intelligence models [15,16,17,18,19]. These algorithms have made consistent the detection of landmark points and the analysis of measurement values possible for less-experienced clinicians. Among the methods for constructing an artificial intelligence system, the machine learning method is one that forms a rule by repeatedly learning input and output values. This is similar to how humans learn rules through repetitive learning, but computers can implement these rules remarkably fast. There have been many studies using various AI models in orthodontics. In the extraction decision problem, there have been studies that applied machine learning models to construct neural networks using landmark measurements to predict outcomes. These studies have drawbacks. To apply the neural network model, they needed to manually measure the landmark points, calculate the measurements, and then put them in the input node. Another downside is that some information may be missing. Only the specific measurements selected by the person who configured the neural network may be entered.

In addition, a previous study was conducted to diagnose with an X-ray image using convolutional neural networks (CNNs), going beyond analysis using measured values [20,21]. A previous study already demonstrated an accuracy that exceeded methods that used measured values. This reduced errors arising from the accuracy of a clinician’s tracing skills or the accuracy of an automatic artificial intelligence landmark-detection model, and allows for easy and intuitive decision-making for less experienced clinicians.

In a previous study, the difference in the performance of the diagnostic model was compared with the difference in the CNN model and the reasons for these differences were analyzed. One of the reasons was the complexity of the CNN model, which possibly degraded its diagnostic performance using medical images. In a previous study, a Modified-AlexNet with moderate complexity exhibited the best performance [21,22]. For a more precise analysis of the conclusions of these previous studies, the same model with neural networks of different depths should be studied; therefore, the differences in the predictive ability according to the depth of the neural network can be compared without the confounding effect of any other differences in the models.

Therefore, this study aimed to investigate the relationship between image patterns in cephalometric radiographs and the need for orthognathic surgery, and report on a method for improving the accuracy of predictive models according to the depth of the neural network. The null hypothesis is that there is no difference in predictive ability depending on the depth of the neural network.

## 2. Materials and Methods

In this study, 960 patients who visited the Seoul National University Dental Hospital for orthodontic treatment were included. All patients had radiographs and clinical photographs taken for routine clinical examination and all patients were diagnosed by an orthodontic specialist. The patient group included 640 patients who needed non-surgical orthodontic treatments and 320 patients who needed orthognathic surgery. The study was conducted according to the guidelines of the Declaration of Helsinki and approved by the Institutional Review Board of Seoul National University Dental Hospital (ERI21009).

**Figure 1:**
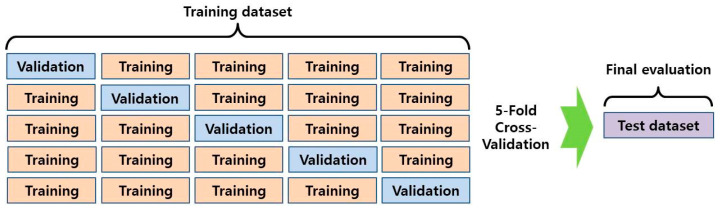
The overview of the 5-fold cross-validation used in this study.

Of the total patients, data of 150 patients were classified as the test set and excluded from training. The data of the remaining 810 patients were split into five groups and 5-fold cross-validation was performed (Figure 1) [23]. At each step, a model was constructed and its performance was evaluated by calculating its success rate for identifying the need for surgery in the training, validation, test, and total sets.

The landmarks of the patients’ cephalometric radiographs were automatically detected with a software program using a gradient boosting algorithm (WebCeph, AssembleCircle, Seoul, Korea). WebCeph has been used for landmark detection in several studies [24,25]. The minimum box, including all the landmarks, was selected with an external margin of 5% and the lower part of the box was selected by square cropping. The image was resized to 256 × 256 pixels.

**Figure 2:**
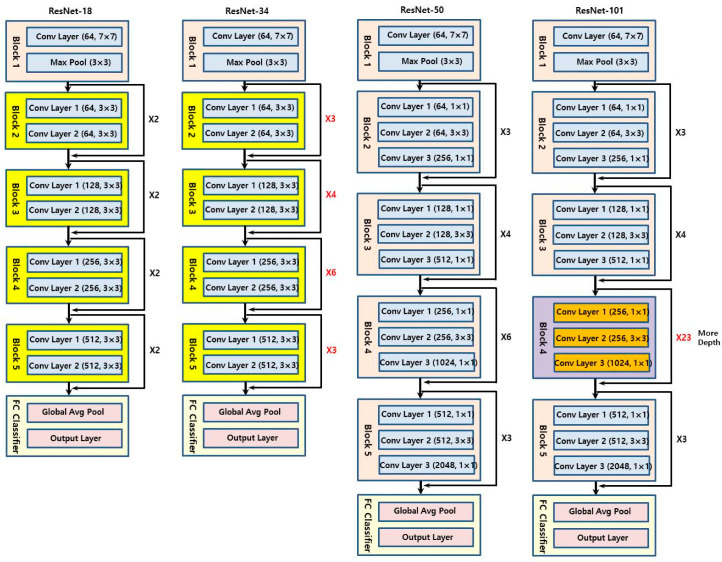
Schematic diagrams of the deep learning models used in this study: ResNet-18, ResNet-34, ResNet-50, ResNet-101.

**Figure 3:**
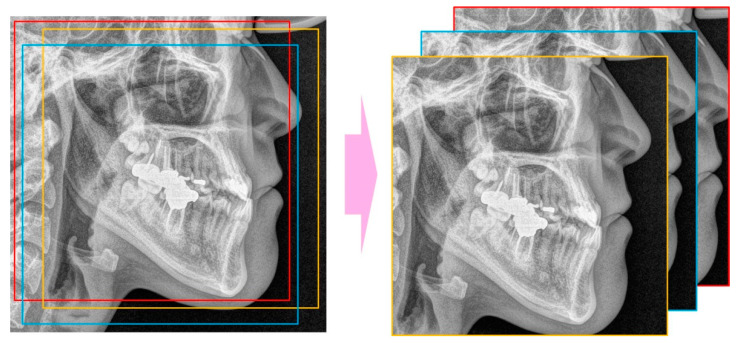
Random cropping to get 224 × 224 images from 256 × 256 image.

The CNN models used were ResNet-18, 34, 50, and 101 (Figure 2) [26]. The number was determined by the difference in the depth of the blocks that constitute the model. As ResNet models use an image of 224 × 224 pixels as the default input image, the input image was selected by randomly cropping the 256 × 256 image (Figure 3).

Through this process, overfitting was prevented, and random horizontal flipping, dropout, and batch normalization were performed for the same reason [27]. The number of epochs was set to 150, and the batch size of the training and validation sets was set to 32. A stochastic gradient descent (SGD) optimizer was used with the learning rate set to 0.002, the decay to 1 × 10^−6^, and the momentum to 0.9 [28,29]. The learning rate was adjusted by a factor of 0.1 if the validation loss did not improve above 1 × 10^−6^ during 30 epochs. Training accuracy, training loss, validation accuracy, and validation loss were checked, and the predictive ability was measured for each of the four models. Subsequently, the accuracy, sensitivity, and specificity of each model were measured and compared. The receiver operating characteristic (ROC) curves and the area under the ROC curve (AUC) for each model were calculated. All models were trained on a 64 bit Windows 10 system, with 32 GB memory and an NVIDIA GeForce RTX GPU. Implementation of the deep learning models was performed using the Python Keras library and TensorFlow backend engine.

## 3. Results

### 3.1. Clinical and Demographic Characteristics of the Subjects

The patients consisted of 468 men and 492 women with an age range from 15 to 37 years (mean age of 24.6 years). There were 640 patients (mean age 23.7 years) who needed orthodontic treatment only and 320 patients (mean age 26.3 years) who needed orthognathic surgery treatments. The clinical characteristics of the dataset used in this study are summarized in Table 1.

### 3.2. Prediction Performance

**Figure 4:**
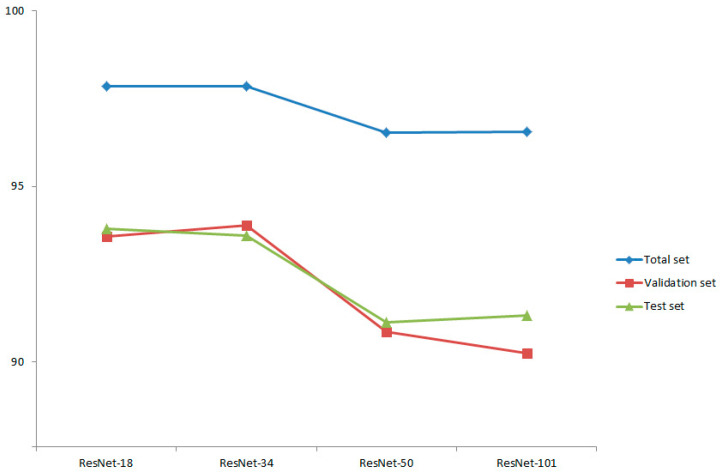
Prediction accuracy of the four models: success rate of total set (blue line), validation set (red line), and test set (green line).

The results of this study showed that the average success rate for diagnosis of orthognathic surgery for the ResNet-18 model was 99.86% in the training dataset, 93.58% in the validation dataset, and 93.80% for the test dataset; it had a total predictive ability of 97.85% for the total dataset. The Resnet-34 model had an accuracy of 99.81%, 93.89%, and 93.60% in the training, validation, and test datasets, respectively, and a total predictive ability of 97.84% for the total dataset. The Resnet-50 model’s accuracy was 99.21% in the training dataset, 90.86% in the validation dataset, and 91.13% for the test dataset; it had a total predictive ability of 96.54% for the total dataset. The Resnet-101 model’s accuracy was 99.34%, 90.25%, and 91.33% in the training, validation, and test datasets, respectively, with a total predictive ability of 96.55% for the total dataset (Figure 4). The models of ResNet-18 and 34 showed higher prediction performance than the ResNet-50 or 101 models. We rejected the null hypothesis that there is no difference in predictive ability depending on the depth of the neural network.

### 3.3. Screening Performance

**Figure 5:**
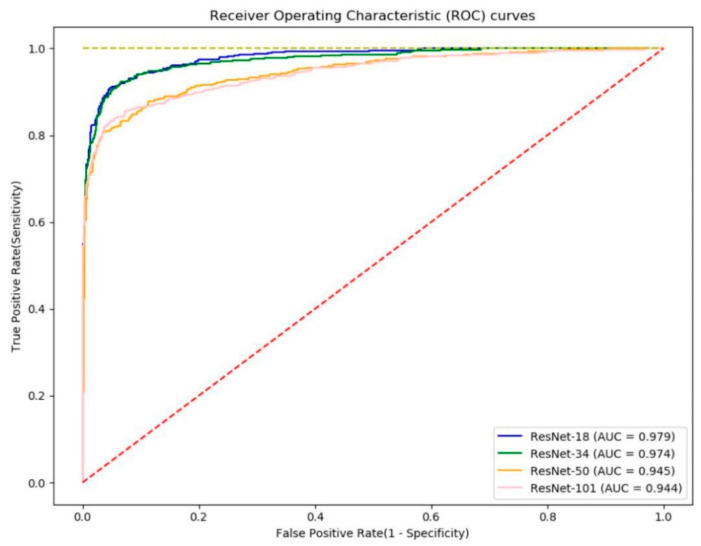
The receiver operating characteristic (ROC) curves of the four models: ResNet-18 (blue line), ResNet-34 (green line), ResNet-50 (orange line), and ResNet-101 (pink line).

**Figure 6:**
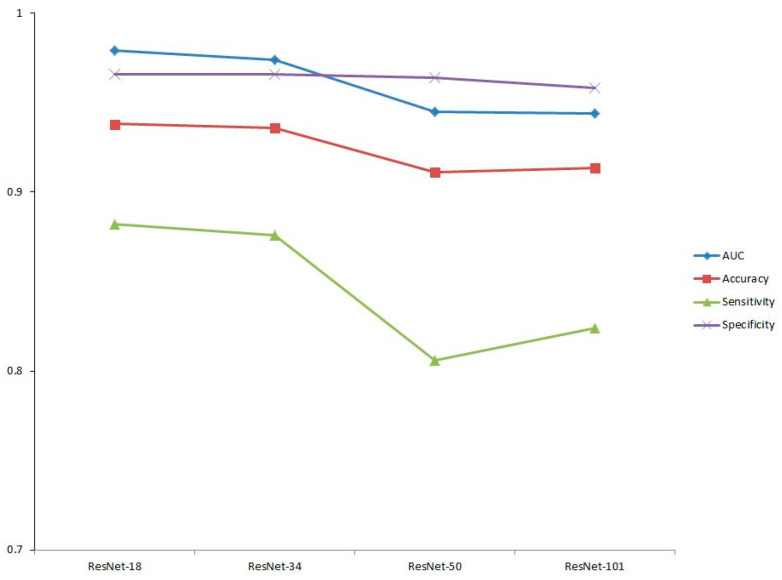
Screening performance of four models: AUC (blue line), accuracy (red line), sensitivity (green line), and specificity (purple line).

Figure 5 shows the ROC curves. The ROC curve is a graph showing the performance of the model through the relationship between the true positive rate and the false positive rate at all classification thresholds. AUC refers to the area under the ROC curve. AUC values range from zero to one. A model with 100% incorrect prediction has an AUC of 0.0 and a model with 100% accurate prediction has an AUC of 1.0. The AUC is not an absolute value, but rather a measure of how well the prediction is evaluated. The AUC measures the predictive quality of a model regardless of which classification threshold is selected. In screening performance, based on the AUCs evaluated for sensitivity and specificity, ResNet-18 had the best performance at 0.979, followed by ResNet-34 at 0.974, ResNet-50 at 0.945, and ResNet-101 at 0.944 (Table 2). Figure 6 shows the screening performance of the four models used in this study. When determining the overall performance, the difference in specificity was not large, but there was a difference in sensitivity that led to the difference in accuracy.

## 4. Discussion

Much of the existing AI research related to orthodontic diagnosis was performed by selecting landmark points and calculating measurements. Artificial neural network (ANN) machine learning (ML) is a representative method of artificial intelligence and is used to guess and approximate a veiled function that depends on many input values. This method can be affected by the input of the measured value. In the process of detecting landmark points, errors can occur. In addition, there is a disadvantage: overfitting is likely to occur when measured values with similar meanings are input into a machine learning model.

The deep learning algorithm is an algorithm that extracts features of an image using a convolutional filter and a pooling layer, and analyzes a pattern in them. Many deep learning models have been refined and developed based on filter sizes, types, locations, combinations, and different ideas. Deep learning is an advanced form of the existing ANN, made possible due to the development of computing ability and easier access to big data. The convolutional neural network is a deep neural network, with multiple hidden layers, which has a structure suitable for learning 2D image data. The cephalograph or clinical photo image can be used as an input value. If the diagnostic image data are analyzed using deep learning, it is expected that the empirical knowledge gained from viewing the image data can be better reflected.

The null hypothesis was rejected. For the same CNN model, the ResNet-18 and ResNet-34 models performed better than the ResNet-50 and ResNet-101 models. The latter number indicates the depth of the residual blocks [26]. In general, deep learning performance degrades when the depth of the network increases overfitting becomes severe, backpropagation is poorly performed, and the feedback of the result does not properly affect the initial weight value [27,30]. The ResNet model was developed to address this problem. The most prominent feature of the ResNet model is that by adding the initial value to the result obtained by passing through the filter of the convolutional neural network, the input value can be well-reflected even if the network is deep; therefore, the weight can be properly adjusted. This results in improved performance despite the network’s tremendous depth.

**Figure 7:**
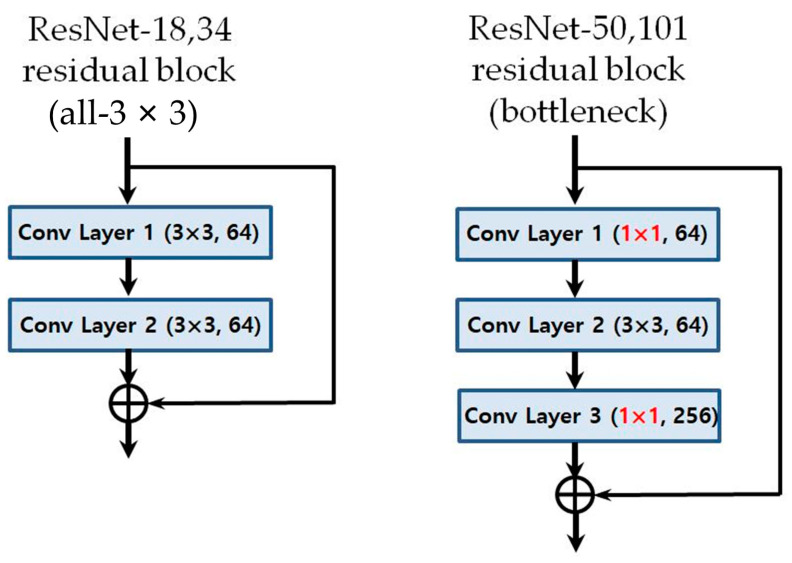
Comparison between models’ residual block structures: 3 × 3 structure for ResNet-18 and 34 and bottleneck structure for ResNet-50 and 101.

In addition, in the case of ResNet-50 and 101 models, which have deeper neural networks than those of ResNet-18 and 34 models, the biggest difference is that the residual block has a bottleneck structure [26]. Unlike the ResNet-18 and 34 models that pass through the 3 × 3 filter twice, ResNet-50 and 101 models are called bottleneck structures because they sequentially pass through a 1 × 1 convolution filter, a 3 × 3 filter, and again through a 1 × 1 filter (Figure 7). This structure is adopted because as the network deepens, the number of weights increases tremendously and the burden of computation increases. By adopting a bottleneck structure, the speed of calculation increases by reducing the amount of computation required without causing a loss of key information. The deeper model showed better performance in the ImageNet classification than the existing ResNet-18 and 34 models and ResNet-50 is one of the most popular state-of-the-art models [31].

Unlike the results of the ImageNet classification, the experimental results of this study showed that the ResNet-18 and 34 models produced better performance than the ResNet-50 and 101 models. This means that unlike the ImageNet model, which has to classify thousands of kinds of images, in a relatively simple model for identifying the need for orthognathic surgery, excessive complexity may degrade performance [31]. In the ILSVRC, it was able to learn based on millions of data, but with medical data, it is not easy to obtain a large enough data size. This study provides strategies to use when learning with a limited amount of data.

In addition, we showed that the linear structure provides better performance than the bottleneck structure, which is advantageous for capturing the characteristics of an image. This shows that a linear structure can be better for prediction regarding the need for orthognathic surgery as it allows a comprehensive judgment based on the entire image, unlike the image classification problem in which a specific area is identified to determine classification. In other words, minimizing the distortion or loss of information can be more beneficial for prediction and confirms that the neural network should not be too deep to use a linear structure effectively.

These results are similar to those of a previous study in which a Modified-AlexNet, a relatively simple model, produced superior performance [21,22]. This confirms that structural differences in artificial intelligence models can lead to differences in predictive ability. This study suggests that prediction using medical images may be better with an artificial intelligence model that contains complete information and a neural network of appropriate depth. Therefore, this study proposes a new direction of research focused on model structure for the development of artificial intelligence models for prediction using medical images.

A limitation of this study was that it was conducted at a single center; if the analysis was conducted with multi-center data, it may have helped create a more general model. In future research, it would be beneficial to employ multi-center data and thereby improve the model’s performance. However, it is a strength that a more generalized model was created and that its performance was analyzed using a larger sample size than previous studies.

The decision regarding the treatment plan reflects the clinician’s experience and preferences. There is no right answer with a treatment plan. The purpose of this study was to create an artificial intelligence system that could imitate the philosophy and decisions of experienced professionals rather than finding the right answer. We analyzed and evaluated the ability, according to the depth and structure of a neural network, of models to predict the need for orthognathic surgery. If a clinician performs tracing of a landmark point on a cephalogram, the precision or the measurements value may vary depending on the clinician’s ability. Diagnosis by the image reduces such inconsistencies. In addition, with the entire image, information can be considered and it reduces the likelihood of the loss of information. This paper provides suggestions on the characteristics of an artificial intelligence model for prediction using medical images.

## 5. Conclusions

The difference in several models’ ability to diagnose whether to conduct orthognathic surgery was analyzed and evaluated according to the depth and structure of their neural networks. The ResNet-18 and 34 models attained 93.80% and 93.60% success rates, respectively, in the test set; this confirmed the performance superiority compared with that of ResNet-50 and 101 models, which showed rates of 91.13% and 91.33%, respectively.

## Figures and Tables

**Table 1:** Demographic characteristics of the samples used in this study.

	Orthodontic Treatment	Orthognathic Surgery	Total
Number of patients	640	320	960
Number of men/women	311/329	157/163	468/492
Mean age (SD), years	23.7 (5.3)	26.3 (4.2)	24.6 (4.9)

SD, standard deviation.

**Table 2:** Screening performance of the four models used in this study.

Model	AUC (95% CI)	Accuracy (95% CI)	Sensitivity (95% CI)	Specificity (95% CI)
ResNet-18	0.979 (±0.008)	0.938 (±0.014)	0.882 (±0.021)	0.966 (±0.010)
ResNet-34	0.974 (±0.009)	0.936 (±0.015)	0.876 (±0.021)	0.966 (±0.010)
ResNet-50	0.945 (±0.014)	0.911 (±0.017)	0.806 (±0.027)	0.964 (±0.011)
ResNet-101	0.944 (±0.014)	0.913 (±0.017)	0.824 (±0.026)	0.958 (±0.012)

AUC, area under the curve; CI, confidence interval.

## Data Availability

The data underlying this article will be shared upon reasonable request from the corresponding author.
